# Electrocatalysts for Hydrogen Evolution in Alkaline Electrolytes: Mechanisms, Challenges, and Prospective Solutions

**DOI:** 10.1002/advs.201700464

**Published:** 2017-11-10

**Authors:** Nasir Mahmood, Yunduo Yao, Jing‐Wen Zhang, Lun Pan, Xiangwen Zhang, Ji‐Jun Zou

**Affiliations:** ^1^ Key Laboratory for Green Chemical Technology of the Ministry of Education Chemical Engineering and Technology Tianjin University Tianjin 300072 China; ^2^ Collaborative Innovative Center of Chemical Science and Engineering (Tianjin) Tianjin 300072 China; ^3^ School of Engineering RMIT University 124 La Trobe Street 3001 Melbourne Victoria Australia

**Keywords:** alkaline electrolytes, electrocatalysts, electrochemical materials, hydrogen evolution reaction

## Abstract

Hydrogen evolution reaction (HER) in alkaline medium is currently a point of focus for sustainable development of hydrogen as an alternative clean fuel for various energy systems, but suffers from sluggish reaction kinetics due to additional water dissociation step. So, the state‐of‐the‐art catalysts performing well in acidic media lose considerable catalytic performance in alkaline media. This review summarizes the recent developments to overcome the kinetics issues of alkaline HER, synthesis of materials with modified morphologies, and electronic structures to tune the active sites and their applications as efficient catalysts for HER. It first explains the fundamentals and electrochemistry of HER and then outlines the requirements for an efficient and stable catalyst in alkaline medium. The challenges with alkaline HER and limitation with the electrocatalysts along with prospective solutions are then highlighted. It further describes the synthesis methods of advanced nanostructures based on carbon, noble, and inexpensive metals and their heterogeneous structures. These heterogeneous structures provide some ideal systems for analyzing the role of structure and synergy on alkaline HER catalysis. At the end, it provides the concluding remarks and future perspectives that can be helpful for tuning the catalysts active‐sites with improved electrochemical efficiencies in future.

## 1 Introduction

Hydrogen has been referred as fuel of future with water as an oxidation product, no carbon and higher enthalpy of combustion than any other chemical fuel.^1^, ^2^, ^3^ Although extensive research has been carried out to produce the hydrogen fuel, still sustainable production of hydrogen is a great challenge.^4^ At present about 44.5 million tons of hydrogen is produced worldwide from various sources, e.g., steam methane reforming, coal gasification, and water electrolysis. The former two produce 96% while the later one produce only 4% of total hydrogen, which is mainly used for industrial applications, i.e., oil reforming and ammonia production for fertilizer.^5^, ^6^ The production of hydrogen through hydrocarbon cracking results in impure gas and toxic effluents to environment that severely affect the climate.^7^ The hydrogen production through electrocatalytic water splitting has been proved as an alternative clean and sustainable fuel to finite fossil fuels.^8^, ^9^, ^10^ However, construction of specified infrastructure for the storage and delivery of hydrogen poses a significant challenge in the hydrogen highways. The deployment of “Plug and Play” electrolyzer makes the market‐driven installations of refueling stations possible, but the use of highly acidic proton exchange membrane (PEM) systems increase its cost and stability concerns.^11^, ^12^ Moreover, these systems use platinum group metals (PGMs) based catalysts for hydrogen evolution reaction (HER, one of the most fundamental reaction for hydrogen production), and operate under highly corrosive conditions (strong acids) which further increases the cost and safety concerns of overall setup.^13^ Therefore, development of alternative catalysts and reaction medium is dire need of time to fulfill the demands of future energy.^14^, ^15^ The HER setup under alkaline conditions present an attractive substitution that not only increase the stability of PGMs‐based water electrolyzer but also open the possibility of hunting transition metal based catalysts. However, the decreased hydrogen kinetics is a great challenge associated with alkaline‐HER technology.^16^, ^17^

The adsorption energy of the catalysts constitutes a key parameter in determining the conversion efficiency and stability in an electrolyte solution. A moderate adsorption energies for water and hydrogen on the active sites with low attraction to hydroxyl ions is required to proceed the reaction in alkaline medium at lower overpotentials, better efficiencies and stability.^18^ The theoretical studies have shown that the activity trends in acidic medium are concluded on the basis of hydrogen adsorption (H_ad_) at active sites but in alkaline mediums it is controlled by the delicate balance among three important descriptors; (i) the H_ad_ on the surface of catalyst, (ii) the prevention from hydroxyl adsorption (OH_ad_) referred as poisoning of active sites, and (iii) the energy requires to dissociate water molecules.^18^, ^19^, ^20^, ^21^ To confirm the importance of third descriptor, Markovic and co‐workers carried out a series of experiments by modifying the surface of metal with water dissociation catalyst such as Ni(OH)_2_, where Ni(OH)_2_ enhanced the dissociation of water molecules to produce H atoms which can then be collected by the metal active centers and recombined into hydrogen.^22^ Thus, to overcome this additional energy and faster the reaction kinetics, new electrocatalyst that have the ability to dissociate water molecules and moderate affinity for H_ad_ as well as its recombination to yield hydrogen gas need to be developed.^11^, ^23^, ^24^, ^25^

Unfortunately, till now only PGMs have shown considerable affinity to catalyze water in alkaline medium at moderate overpotentials, while that of transition metals (TMs) need high overpotentials.^26^, ^27^ Therefore, to‐date, the PGMs‐based catalysts have been predominantly used to generate H_2_ from alkaline water at industrial scale.^28^, ^29^ However, to find alternative HER catalysts to produce hydrogen from electrocatalytic water splitting on global scale in alkaline medium, a world‐wide aggressive research has been undertaken recently. A range of catalysts have been investigated from micro‐ to nano‐ and solo‐ to heterostructured based on earth abundant metals, such as heteroatom‐doped carbon and TMs‐based oxides, sulfides, phosphides, sulfophosphides, and their alloys.^30^, ^31^, ^32^, ^33^, ^34^, ^35^ However, catalyst optimizations are facing great difficulties in characterizing, identifying, and controlling the active sites where catalysis occurs.^36^ Besides the activity and active sites issues, the stability of the catalysts constitutes another concern for earth‐abundant catalysts. For instance, the nonprecious metal catalysts can undergo segregation due to metal dissolution as well as change in surface chemistry due to varying composition.^37^ Thus, the development of integrated electrocatalysts for alkaline HER are highly required to overcome the issue of poor reaction kinetics and stability. These issues can be resolved by tuning the surface chemistry by modifying the electronic structure, composition, morphology and porosity/active surface area. On the other hand molecular complexes based on porphyrin and corrole architectures, such as nickel porphyrins, copper corroles, etc. have also shown their possible use.^38^, ^39^, ^40^, ^41^, ^42^ It is worth mentioning that recently designed Co corrole complex 5,15‐bis‐(pentafluorophenyl)‐10‐(4)‐(1‐pyrenyl)phenyl containing a triphenylphosphine axial ligand by Cao and co‐workers is the first example of a molecular HER catalyst that is active and stable in a wide pH range (0–14).^43^

Currently a plenty of catalysts have been developed that have comparable or even better catalytic activity than the benchmark PGMs‐based catalysts.^44^ Recently, it is also found that few catalysts have ability to perform better in alkaline medium than the acidic one, e.g., Hu and co‐workers prepared NiCo_2_P*_x_* as an active catalyst that can achieve current density of 10 mA cm^−2^ at 58 and 104 mV in alkaline and acidic mediums, respectively, where Ni modified the Co electronic atmosphere.^45^ Thus, demonstrates that if we could effectively play with the catalyst electronic structure, morphology and composition, it is possible to achieve the HER catalysis in alkaline medium with same pace as in acidic medium, which is also one motivational demand to us in reviewing this research area. Although several good review articles have been published on acidic or/and wide pH HER catalysis, however, a comprehensive review on HER catalysis in alkaline environment is still missing and is need of time given the large interest of researchers toward alkaline HER.

This review intends to summarize the recent developments in electrochemistry and synthesis of advanced electrocatalysts to overcome the kinetics issues of alkaline HER. This review will also discuss the possible modifications in morphologies and electronic structures of electrocatalysts to tune their active sites for stable performances as efficient catalysts for HER. First we will explain the fundamentals and electrochemistry of HER in alkaline medium. Then we will outline the requirements for an efficient and stable catalyst, challenges with HER in alkaline medium and limitations of electrocatalysts along with prospective solutions. Further we will describe the synthesis methods for advanced nanostructures based on carbon, noble, and inexpensive metals, and their heterogeneous structures as ideal systems for analyzing the role of structure and synergy on alkaline HER catalysis. At the end, we will list the concluding remarks and future perspectives that may lay down the foundations to improve the electrochemical efficiencies of catalysts by tuning the chemistry of active sites.

### 2 Importance and Fundamentals of HER in Alkaline Medium

The hydrogen has been taken as one of the most attractive alternatives to the rapidly exhausting traditional fossil fuels because of its environment benignity and recyclability.^46^ The electrocatalytic water splitting has been proved as an efficient and clean technology, compared to other hydrogen production strategies as it provides an easy opportunity for coupling with other renewable energy, e.g., solar and wind, etc.^4^ In this regard, alkaline water electrolysis is of a prime importance because it is one of the most widely used technology in the industry where it can play a key role to treat the excreted alkaline water, e.g., water‐alkali and chlor‐alkali electrolyzers.^6^ These are also high energy consuming technologies: water‐alkali and chlor‐alkali electrolyzers consumed up to 25–30% of the total electrical energy consumptions in the United States.^37^ Besides, the HER in alkaline medium is also a typical reaction for other fundamental sciences to explore new insights. However, very few electrocatalysts have been reported so far that have the ability to compete with Pt in alkaline medium in terms of reaction efficiency.^47^ Therefore, the reaction mechanism needs to be explored and basic laws of electrode kinetics should be verified that may lay down the foundation for researchers to hunt new and efficient electrocatalysts.

Although a bunch of active electrocatalysts toward HER in acidic medium have been reported that exhibit superior activities. But the operational condition of acid based PEM cells inevitably result in the vaporization of electrolyte or strong acidic fog, resulting in the corrosion of electrolytic cell as well as contaminates the as‐produced hydrogen gas.^16^ On the contrary, production of lesser vapors under high temperature working conditions is the advantage of alkaline electrolytes due to their lower vapor pressure, which helps in the production of pure hydrogen gas.^37^ Furthermore, alkaline mediums also provide better stability to non‐noble metals by avoiding their corrosion and dissolution, hence resulting in prolonged catalysis and make it an attractive alternative over the acidic catalysis.^12^

It is well‐known that the water electrolysis in different electrolytes follow different pathways, like large number of available protons promote HER catalysis in acidic electrolytes, while the HER is comparatively harder to achieve at low overpotentials in alkaline medium.^48^ The HER in alkaline solution needs extra energy to produce the protons by breaking the water molecule, consequently influencing the overall reaction rates.^13^, ^22^ Various theoretical and experimental studies have tried to enlighten the HER mechanisms in alkaline medium and found that HER involves three step under alkaline medium: (i) the Volmer step, (ii) water dissociation, and (iii) creation of a reactive hydrogen intermediates (2H_2_O + M + 2e^–^ → 2M − H_ad_ + 2OH^−^, M denotes catalyst surface here) that is different from the mechanism in acidic medium (2H_3_O^+^ + 2e^−^ + M → 2M – H_ad_ + 2H_2_O), which normally follow either the Heyrovsky pathway or the Tafel recombination step.^37^ Similarly, the HER pathway in alkaline medium can also follow the Volmer–Heyrovsky step or Volmer–Tafel step as shown in Equations (1)–(3). In contrast to the acidic conditions where inimitably water is electrochemically dissociated into adsorbed OH^−^ and H* species in the Volmer step,^16^ in alkaline medium the catalyst requires to break the stronger covalent H—O—H bond prior to adsorb H*, rather than the dative covalent bond of hydronium ion that could easily be pursued by the reduction of H_3_O^+^.^49^ Considering theoretical predictions and experimental results, Yang and co‐workers have reported that due to lower HER kinetics high loadings of Pt or Pt/C are required to run PEM systems which significantly increases the cost.^50^ Thus, a deep understanding of alkaline HER catalysis is highly desirable that can guide us toward developing better catalysts to make it economically favorable.

Utilizing the Tafel slope values given below in Equations (1)–(3) as reference, one can analyze the reaction rout on a specific catalyst in alkaline electrolytes

(1) $$H_{2}O+e^{-}\to H^{*}+{\mathrm{HO}}^{-}$$

Volmer (120 mV dec^−1^)

(2) $$H_{2}O+e^{-}+H^{*}\to H_{2}+{\mathrm{HO}}^{-}$$

Heyrovsky (40 mV dec^−1^)

(3) $$H^{*}+\;H^{*}\;\to H_{2}$$

Tafel (30 mV dec^−1^)

All the above listed reaction phenomenon strongly depend on the inherent surface chemistry and electronic structure of catalyst. A lot of efforts have been put forward to develop new alkaline medium active catalysts but still several key questions concerning the activity of these catalysts are unanswered. As an example, why the activities of catalysts are 2–3 folds lower in alkaline medium than in the acidic medium, even the best performing Pt.

## 3 Challenges Associated with HER in Alkaline Medium

**Figure 1:**
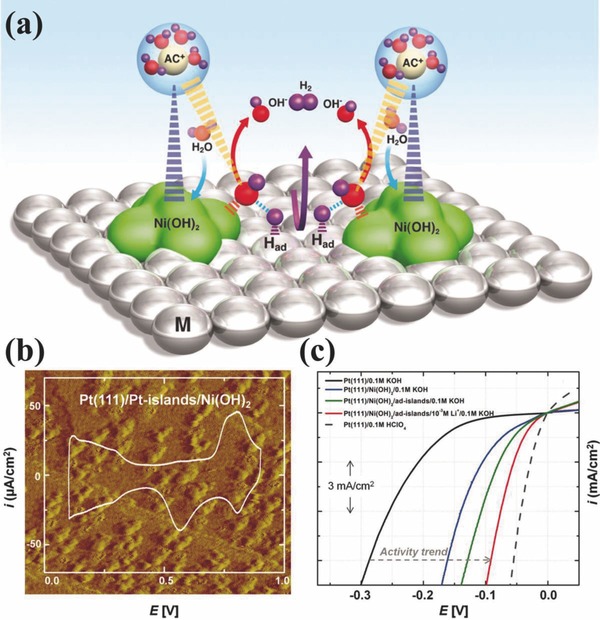
a) Schematic representation of water dissociation, formation of M‐H_ad_ intermediates, and subsequent recombination of two H_ad_ atoms to form H_2_ (magenta arrow) as well as OH^−^ desorption from the Ni(OH)_2_ domains (red arrows) followed by adsorption of another water molecule on the same site (blue arrows). Water adsorption requires concerted interaction of O atoms with Ni(OH)_2_ (broken orange spikes) and H atoms with Pt (broken magenta spikes) at the boundary between Ni(OH)_2_ and Pt domains. The Ni(OH)_2_‐induced stabilization of hydrated cations (AC^+^) (broken dark blue spikes) likely occurs through noncovalent (van der Waals type) interactions. Hydrated AC^+^ can further interact with water molecules (broken yellow spikes), altering the orientation of water as well as the nature and strength of interaction of the oxide with water. b) STM image (60 nm × 60 nm) and CV trace of the Ni(OH)_2_/Pt‐islands/Pt(111) surface. Clusters of Ni(OH)_2_ in the STM image appear ellipsoidal with particle sizes between 4 and 12 nm. c) Comparison of HER activities with Pt(111) as the substrate. Incremental improvements in activities for the HER in 0.1 m KOH from the unmodified Pt(111) surface are shown for the hierarchical materials [ad‐islands, Ni(OH)_2_, and their combination] as well as the double layer (addition of Li^+^ cations). The activity for the unmodified Pt(111) surface in 0.1 m HClO_4_ is also shown for reference (dashed arrow shows the activity trend). Reproduced with permission.^13^ Copyright 2011, AAAS.

The answer of the question why the high performing acidic medium based catalysts are not efficient in the alkaline electrolytes lie in the requirements of high overpotentials to initiate the catalysis along with the poor power efficiencies in alkaline medium. The reaction chemistry suggests that there is an additional energy barrier required that needs to be overcome by the catalyst to continue the electrocatalytic hydrogen production. It has been experimentally proved by developing hybrid catalyst that has an active component for water dissociation. It is found that incorporation of water active catalyst can increase the HER activity of its counterpart (HER active catalyst).^22^ For example Markovic group has developed the Ni(OH)_2_ decorated Pt catalyst to improve the HER activity of Pt by lowering the energy barriers for water dissociation as shown in **Figure** **1**.^13^ Such a bifunctional catalyst can increase the Pt activity by 8 folds (Figure 1c) by working through a simple mechanism. A water dissociation happens at the edges of Ni(OH)_2_ clusters and produces the hydrogen intermediates that adsorb on the surface of neighboring Pt active sites and recombine to yield molecular hydrogen. To further confirm the requirement of high energy for additional water dissociation step and eliminate the confusion of Pt, hybrid catalysts based on cost‐effective metals, e.g., Ni, Cu, Ag have been designed and found that after surface modifications by Ni(OH)_2_ the performance of these metals also approach to their activity in acidic medium.^22^ Although this method has enhanced the activity of PGMs and cost effective metals in alkaline medium but have several reservation for practical applications likewise complex synthesis methods, hard to control the interface and stability concerns on longer use.

Besides the water dissociation, the poor water binding energies than the hydronium ion is another challenge to produce M—H bond in alkaline medium. Recently, Baek and co‐workers have prepared the Ru nanoparticles (NPs) decorated on nitrogenated holey 2D carbon structure (C_2_N) and hybrid comes up with exceptional HER activity by achieving the current density of 10 mA cm^−2^ at the over potential of 17 mV which is even better than the commercial Pt/C.^18^ The theoretical studies suggested that when Ru is placed in the holes of C_2_N, the Ru–H_2_O binding energy increases significantly, which results faster water adsorption and accelerates Volmer step. Similarly, the poisoning of active sites by the OH^−^ ions also lowers the activity in alkaline medium by limiting the number of sites, hence resulting in higher overpotentials. While density functional theory (DFT) studies also demonstrated that the Ru/C_2_N hybrid has low affinity to OH^−^ ions, hence making it better catalyst for alkaline HER. All these factors contribute toward a complex reaction mechanism on the surface of catalyst surface in alkaline medium and is not as simple as in acidic media where the HER activity can be correlated with exchange current densities in terms of catalyst ability to bind the hydrogen.^5^ Moreover, the unprecedented coverage of surface by spectator species even in the HER potential region further limits reasonable prediction about exact rate controlling factors and their relationships. However, few theoretical studies have shown that the catalyst performances in alkaline medium are predominantly controlled by two factors, i.e., water dissociation and hydrogen binding energy. Thus, any catalyst have better ability to dissociate the surface adsorbed water molecules and good binding capability for as produced hydrogen species will result better HER catalysis in alkaline media.^51^ Furthermore, Yan and co‐workers correlated the exchange current density with the hydrogen binding energy supported both by theoretical and experimental studies to show the activity trend of different metals in alkaline medium as discussed in the subsequent sections.^20^ Moreover, we will also highlight how the structural and morphological integration of different existing catalyst can improve their activities in the alkaline medium. As Jan Rossmeisl team has proved it theoretically and experimentally by taking the Cu–W alloy as an example which perform better than both of the individual metals due to its modified electronic structure.^52^

## 4 Factors Controlling the Reaction Rate on Catalyst Surface and Materials Designing

PGMs generally exhibit the highest activity for HER not only in acidic but also in alkaline mediums due to their appropriate hydrogen binding energies.^53^ However, Gasteiger and co‐workers studied the HER activities of three monometallic surface, i.e., Pt, Pd, and Ir in both acidic and alkaline mediums by growing corresponding NPs on carbon support and found that the exchange current densities are approximately two orders of magnitude lower in alkaline medium than acidic one, showing poor activities of these metals in alkaline medium.^54^ The lower exchange current densities pointed out that the H‐binding energy could be a suitable descriptor for HER reaction mechanism in both acidic and alkaline mediums. However, the differences in H‐binding energy between alkaline and acidic mediums could not be explained and are still not clear. Furthermore, it has also been found that there are few catalyst that perform better in alkaline medium than the acidic one, although majority of catalysts show better activities in acidic than in the alkaline medium,^18^, ^55^, ^56^, ^57^ suggesting that there are both qualitative and quantitative differences of the reaction mechanisms between these two mediums. Therefore, a thorough understanding of the reaction mechanisms in alkaline medium is significant to ascertain reactive sites, get insight into the reaction mechanism and tailor new materials for HER. Recently, the monocrystalline and polycrystalline PGMs surfaces have served for overall comprehension of the HER process in alkaline medium both by experimental and theoretical studies.^20^, ^58^, ^59^ However, the conclusions from ideal behaviors of PGMs are not fully applicable on low cost catalysts due to their complex electronic structures.

**Figure 2:**
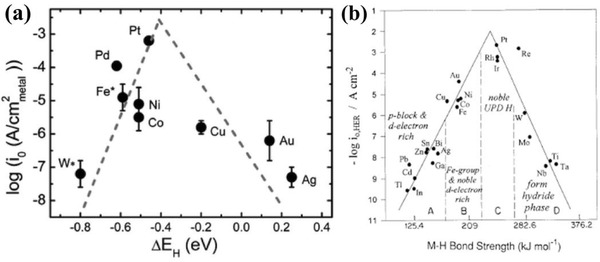
a) Exchange current densities, log(*i*_0_), on monometallic surfaces plotted as a function of the calculated HBE. The *i*_0_s for non‐Pt metals were obtained by extrapolation of the Tafel plots between −1 and −5 mA cm_disk_^−2^ to the reversible potential of the HER and then normalization by the ESAs of these metal surfaces. The dashed lines are guides for the eye. Reproduced with permission.^20^ Copyright 2013, Royal Society of Chemistry. b) Volcano curve for electrocatalysis of the HER at various metals in terms of dependence of log *j*_o_ values on metal‐to‐H bond energy. Reproduced with permission.^21^ Copyright 2000, Elsevier.

The volcano plots have been used as guiding principle to tailor HER catalysts in acidic medium, which predict metals with an optimal H‐binding energy (neither too strong nor too weak) will show highest activity as well as be at the top of volcano such as the PGMs.^60^, ^61^ The optimal H‐binding energy describes neither poor adsorption of the reactants nor difficulty in releasing the final product (**Figure** **2**a).^59^, ^62^ Recently, similar to the case in acidic medium, Yan and co‐workers demonstrated that the relation between HER exchange current density in alkaline medium and H‐binding energy values can be correlated via a volcano type of relationship, supported both by the experimental and DFT studies.^20^, ^58^ Primarily by studying the HER activities on a series of monometallic surfaces through different electrochemical measurements such as linear sweep voltammetry (LSV) on non‐Pt surfaces and cyclic voltammetry (CV) on Pt surfaces, the exchange current densities log (*j*_0_), roughness factors and Tafel slopes were correlated.^20^ Afterward, DFT calculations were applied to calculate the H‐binding energy values via the Vienna ab initio simulation package.^63^ For further exploring the relationship between the HER activity and H‐binding energy, the similar volcano plots was plotted that set HER exchange current densities as a function of calculated H‐binding energy values of these metallic surfaces (Figure 2b).^20^ As shown in Figure 2b, Pt is still at the peak of the plot, and W, Fe, Ni, Co, and Pd are located on the left‐branch for their too strong H‐binding energy, similar to the case in acidic medium. The HER activity in alkaline medium decreases by several orders of magnitude as the H‐binding energy varies from that of Pt in volcano curve, thus strongly suggesting that the H‐binding energy can be a useful descriptor for identifying HER electrocatalysts, by tuning surface chemical properties for an optimal H‐binding energy values to enhance HER activity.^20^ However, Schmickler and co‐workers recently explained that the volcano plots shape may lost its applicability once oxide‐cover on the surfaces of metals such as Mo, Ti, and W are in situ formed since oxide cover lowers the performance.^64^ It was concluded that Sabatier's principle mainly determines the reaction with special exception of Ni and Co as these are 3d metals having compact and low overlapping with hydrogen, makes them good catalysts.^64^ For other metals the reaction rate does not only decrease with highly exothermic hydrogen adsorption, also by its complex pathway as the reaction goes through several intermediate states. So a good catalyst should meet one of these three parameters, i.e., (i) it must obey Sabatier's principle (Δ*G* ≈ 0 at the equilibrium potential), (ii) preferable to have a d‐band to span the Fermi level, and (iii) as the electron transfer from active catalyst (adsorption site) to the proton requires a certain distance (0.5 Å). As a result the catalysts having strong interaction among their d‐band and the 1s orbital of hydrogen will be better.

**Figure 3:**
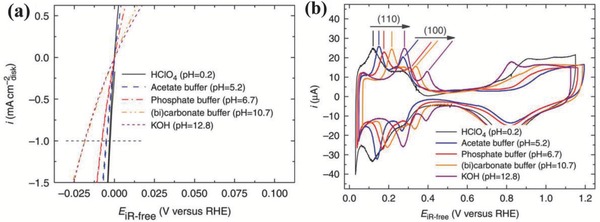
a) HER on Pt in a full range of solution pH. Steady state positive‐going sweeps of HER polarization curves of Pt collected in selected H_2_‐saturated buffered electrolytes. The sweep rate is 10 mV s^−1^ and the rotating speed is 1600 rpm. The polarization curves have been corrected for solution resistance. b) CVs and HBEs of Pt in a full range of solution pH. Steady state CVs of Pt collected in selected Ar‐saturated electrolytes at a sweep rate of 50 mV s^−1^. The CV curves have been corrected for solution resistance. Reproduced with permission.^58^ Copyright 2014, Nature Publishing Group.

Furthermore, the relation between HER activity and experimentally measured H‐binding energy determined by CVs of Pt in pH‐buffered electrolytes (**Figure** **3**) strongly supports the hypothesis that H‐binding energy is more likely the sole descriptor for the HER activities on the surfaces of single metals (Pt) in alkaline environments.^58^ It is also observed that the OH^−^ in the solution phase from alkaline medium largely affect the H‐binding energy by participating in the reaction through adsorption on active surfaces and influencing the HER activity in turn. While, Markovic and co‐workers have raised the question that if the H‐binding energy is the only descriptor for the rate determination of HER, then how it accounts for the phenomenon that catalysts show systematically different activities in alkaline and acidic mediums.^65^ So, three major parameters are identified based on the recent findings that control the HER activity in any aqueous system, i.e., (i) the nature of the proton donor, (ii) the energy of formation of activated complex from proton donor, and (iii) the availability of active sites for the reaction.^65^ In conclusion, the mechanisms of HER activity in alkaline solution are still being debated. It is significant to unravel the fundamentals behind the HER activity by modern techniques, hence require further investigations to design and tailor mono‐ or/and bimetallic materials along with their hetero‐ nanostructures with conductive supports as highly efficient electrocatalysts for alkaline‐HER.

## 5 Overview of Active Electrocatalyst in Alkaline Electrolyte

To date, the efficient HER catalysts for the alkaline medium are much less in number than those for the acidic one. Further, the research focused on developing HER catalysts in alkaline medium with high activity and stability is meaningful and promising for commercial viability. The effective HER catalysts in alkaline medium can be divided into three groups based on the elements used, such as: (i) noble metals and their alloys, e.g., Pt, Pd, Ir, Ru, Ag, etc., (ii) inexpensive transition metal catalysts and their heterogamous nanostructures including Fe, Co, Ni, Mn, Cu, Mo, W, and (iii) nonmetal catalysts comprising of B, C, N, P, S, and their alloys. Herein, the overview of alkaline based catalysts is summarized in three parts based on recent achievements, faced challenges and possible ways to improve their activities. **Tables** **1**, **2**, **3**, **4** are presenting the comparison of catalytic activities of various catalysts toward alkaline HER under different physical parameters.

**Table 1:** Selected summary of the HER performance about noble‐based catalysts in alkaline medium

	Reaction condition				Overpotential [mV]					
Catalyst	Medium	Substrate	Loading mass [mg cm^−2^]	Scan rate [mV s^−1^]	Current density [*j*, mA cm^−2^]	η at the corresponding *j* [mV]	Exchange current density *j*_0_ [mA cm^−2^]	TOFs [H_2_ s^−1^]	Tafel slope [mV dec^−1^]	Ref.
Pt	0.1 m KOH	Pt disk	–	10	1	30	0.62 ± 0.01	–	−113 ± 1	20
Pt–Sm (at 25 °C)	8 m KOH	Pt–Sm electrode	–	0.5	100	366	1.50	–	169	29
Pt–Ho (at 25 °C)	8 m KOH	Pt–Ho electrode	–	0.5	100	414	0.25	–	131	29
Pt–Ce (at 25 °C)	8 m KOH	Pt–Ce electrode	–	0.5	100	390	0.36	–	114	29
Pt/C	0.1 m KOH	GCEa)	0.01	10	5	52	0.23	–	–	68
Pt–Ni–Co (PNCH)	0.1 m KOH	GCE	0.01	10	5	22	0.9	–	–	68
Pt/Fe‐NF	0.05 m KOH	Ni foam	0.1500	20	13.5	0.01	8.538 × 10^−3^	–	59.90	67
Li+/Ni(OH)_2_/Pt	0.1M KOH/LiOH	GCE	≈0.06	50	10	33	–	–	–	13
Pd/C	0.1 m NaOH	GCE	0.016	10	–	–	0.06 ± 0.02 (at 313 K)	–	–	54
Ir/C	0.1 m NaOH	GCE	0.008	10	–	–	0.37 ± 0.12 (at 313 K)	–	–	54
Ru@C_2_N	1 m KOH	GCE	0.285	5	10	17	–	0.75 at 25 mV	38	17
					15	27				
					20	35.5				
Ru/C_3_N_4_/C	0.1 m KOH	GCE	–	10	10	79	–	4.2 at 100 mV	–	44
RuO_2_/Co_3_O_4_	1 m KOH	GCE	0.285	5	10	89	–	–	91	79

^a)^ GCE: glassy carbon electrode.

**Table 2:** Selected summary of the HER performance about single‐non‐noble‐metal‐based catalysts in alkaline medium

	Reaction condition				Overpotential [mV]					
Catalyst	Medium	Substrate	Loading mass [mg cm^−2^]	Scan rate [mV s^−1^]	Current density [*j*, mA cm^−2^]	η at the corresponding *j* [mV]	Exchange current density *j*_0_ [mA cm^−2^]	TOFs [H_2_ s^−1^]	Tafel slope [mV dec^−1^]	Ref.
Ni nanoparticle	1 m NaOH	GCE	0.35	50	10	180	0.191	–	111	75
Ni_3_N nanosphere	1 m KOH	Ni foam	–	–	100	150	–	–	–	92
Co‐NRCNTs	1 m KOH	GCE	0.28	–	1	160	–	–	–	90
					10	370				
CoP/CC	1 m KOH	Carbon cloth	10.3	0.5	10	48	0.76	–	42.6	55
					20	62				
CoP@BCN	1 m KOH	GCE	–	–	10	215	–	–	52	50
					20	302				
CoS_2_ NTAa)/CC	1 m KOH	Carbon cloth	1.2	5	10	193	–	–	88	23
Co_9_S_8_@NOSCb)	1 m KOH	GCE	0.28	5	10	320	–	–	105	130
Mo_2_C/NCFc)	1 m KOH	GCE	≈0.28	10	10	100	–	–	65	99
MoP	1 m KOH	GCE	0.86	2	30	180	0.046	–	48	115
p‐WP_2_	1 m KOH	Carbon fiber paper	∼2	5	10	175	0.17	–	131	31
α‐WP_2_						259	–		165	
β‐WP_2_						277	–		180	

^a)^ NTA: nanotube

^b)^ NOSC: N‐, O‐, and S‐tridoped carbon

^c)^ NCF: carbon microflowers.

**Table 3:** Selected summary of the HER performance about mixed‐non‐noble‐metal‐based catalysts in alkaline medium

Catalyst	Reaction condition				Overpotential [mV]					
	Medium	Substrate	Loading mass [mg cm^−2^]	Scan rate [mV s^−1^]	Current density [*j*, mA cm^−2^]	η at the corresponding *j* [mV]	Exchange current density [mA cm^−2^]	TOFs [H_2_ s^−1^]	Tafel slope [mV dec^−1^]	Ref.
NiCo_2_P*_x_*/CF	1 m KOH	Carbon felt	–	5	10	58	–	0.056 (at 100 mV)	34.3	45
					100	127				
NiFe LDH‐NS @ DGa)	1 m KOH	Ni foam	2	5	20	115	–	–	–	80
		GCE	0.283		10	300				
Fe_0.5_Co_0.5_ @ NC/NCNS	1 m KOH	GCE	0.306	–	10	150	–	–	49.1	33
Fe–CoP/Ti	1 m KOH	Ti foil	1.03	2	10	78	–	–	75	105
MoS_2_@Ni/CC	1 m KOH	Carbon Cloth	7.8	5	10	91	0.807	–	89	106
					20	118				
					100	196				
Ni–Mn_3_O_4_/NF	1 m KOH	Ni foam	–	5	10	91	–	–	110	37

^a)^ DG: defective graphene.

**Table 4:** Selected summary of the HER performance about non‐metal‐based catalysts in alkaline medium

	Reaction condition				Overpotential [mV]					
Catalyst	Medium	Substrate	Loading mass [mg cm^−2^]	Scan rate [mV s^−1^]	Current density [*j*, mA cm^−2^]	η at the corresponding *j* [mV]	Exchange current density [mA cm^−2^]	TOFs [H_2_ s^−1^]	Tafel Slope [mV dec^−1^]	Ref.
C_3_N_4_/FTO	0.1 m KOH	FTOa)	–	10	0.8	300	–	–	120	138
ONPPGC/OCCb)	1 m KOH	Carbon cloth	0.1	2	10	446	–	–	154	140
C_3_N_4_–CNT–CF	1 m KOH	Carbon fiber	≈0.5	5	10	131	0.2767	–	79	141
N‐GMT c)	0.1 m KOH 1 m KOH	GCE	0.452	10	10 10	464 432	–	–	116.7 –	142
N, S‐CNT	1 m KOH	GCE	–	5	5	400	–	–	133	30

^a)^ FTO: fluorine‐doped tin oxide

^b)^ ONPPGC/OCC: nitrogen, phosphorus, and oxygen tridoped porous graphite carbon on oxidized carbon cloth

^c)^ N‐GMT: nitrogen‐doped graphene microtubes.

### 5.1 Nobel Metals and Their Alloys

**Figure 4:**
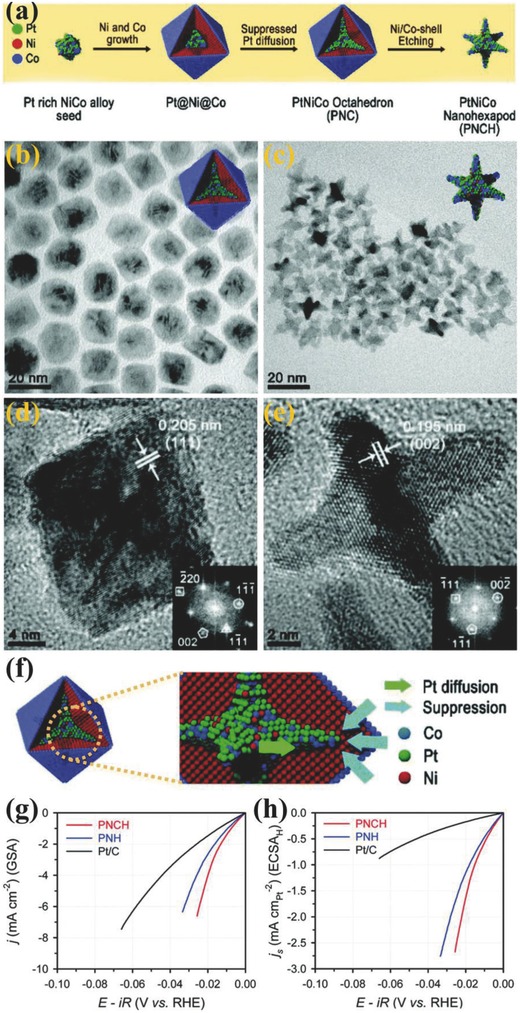
a) Schematic illustration of the formation process of phase segregated Pt–Ni–Co nanostructures. TEM images of b) PNC and c) PNCH with a corresponding inset model. HRTEM with FFT images (insets) of d) PNC and e) PNCH along the 〈110〉 zone axis. The white marks in HRTEM images represent Ni (*d*111 = 0.205 nm) and Pt (*d*200 = 0.195 nm), respectively. f) Schematic illustration the structural evolution of PNC. g) HER polarization curves measured in 0.1 m KOH, h) specific current densities normalized by Pt ECSAH. Reproduced with permission.^68^ Copyright 2016, Royal Society of Chemistry.

The HER catalysis is a surface reaction, so large number of exposed surface sites are important to improve the catalytic activity. Sun and co‐workers have compared the atomically small particle of Pt or their clusters with commercial Pt/C.^66^ They concluded that reduction in size can enhance the Pt activity up to 37 times than commercial Pt/C because of highly exposed surface and involvement of entire active material in catalytic reaction. But the reduction in size should be done with fine control over electronic structure to maintain high stability. Evidently, PGMs still possess an “incomparable” HER activity in alkaline medium as discussed above, which is why researchers focused on the development of their alloys with transition metals to lower their price and enhance their activity by overcoming the inherent limitations.^29^ For example Pt–Re (Re: Sm, Ho, Ce) binary‐alloy based electrocatalysts were designed to improve the stability,^29^ similarly Pt/Fe was electrodeposited on nickel foam for binder free electrocatalyst,^67^ and Pt–Ni–Co ternary alloy nanoframe crystals have been tailored to enhance the efficiency of Pt for HER catalysis in alkaline electrolyte.^68^ Lee and co‐workers explored the effect of Ni and Co coexistence in the Pt ternary alloy by developing PtNiCo alloy nanohexapod (PNCH) dually coated with the Ni@Co shell in a single‐step formation route followed by a selective removal of Ni@Co shell (**Figure** **4**a). During this synthesis, it was observed that the existence of chloride ions mainly control the growth direction and edge chemistry of alloy (Figure 4b–e). The PNCH catalyst exhibited a 10 times superior HER activity to Pt/C, also surpassed binary Pt‐Ni nanohexapods in alkaline medium (Figure 4g,h). Further, the PNCH showed 6 times higher active electrocatalytic surface area than Pt/C, along with an excellent exchange current density (1.4 mA cm_Pt_^−2^). In contrast, the binary PNH deliver comparatively lower exchange current densities of 1.2 and 0.85 mA cm_Pt_^−2^, signifying the promotional role of Co. In addition, it also showed excellent HER durability, attributed to the fact that the Ni and Co contents were leached from [100] branches of the hexapod nanostructure while the overall morphology was preserved during electrochemical reactions.^68^ By an analogous mechanism to Pt/Ni(OH)_2_ as explained above (Figure 1), the faster HER kinetics of Pt–Ni–Co PNCH were attributed to the substantial negative shift in the formation potential of Pt–OH_ad_ and the increase in the charge associated with the H‐binding energy compared to Pt/C, contributed by alloying and bifunctionality.^69^

**Figure 5:**
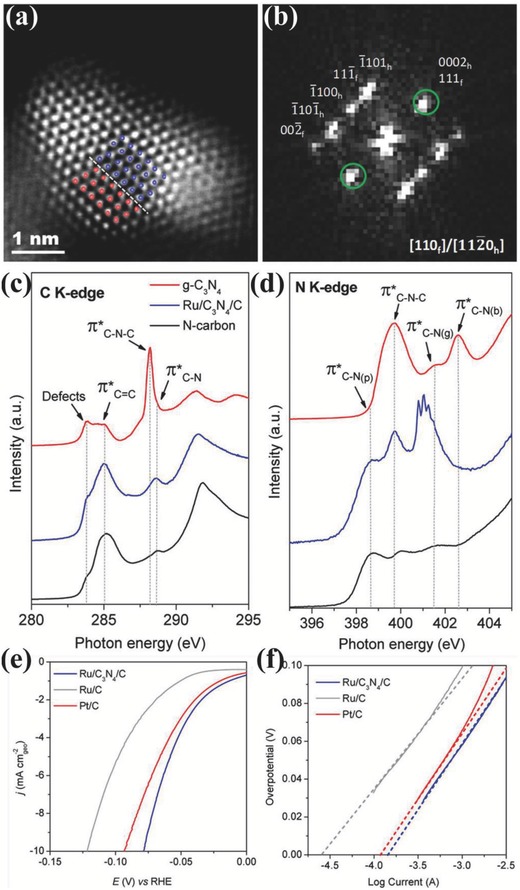
a) HAADF‐STEM images and the b) corresponding FFT patterns of Ru NPs showing mixed fcc/hcp structure. The red and blue dots in panels (a), (c), (e) mark the typical atomic arrangements of fcc and hcp structures along different zone axes. The green circles in panel d inset indicate the shared diffraction plans of the fcc and hcp structures. c) C K‐edge and d) N K‐edge NEXAFS spectra of Ru/C_3_N_4_/C electrocatalyst, pure g‐C3N4, and N‐carbon reference samples. In C Kedge, defects at ≈283 eV in all three materials are assigned to low coordinated carbon atoms at the edges of g‐C_3_N_4_ and N‐carbon moieties. The resonances of π* at 288.2 eV are assigned to C—N—C species in g‐C_3_N_4_, while the resonances of π* at 285.0 eV and π* at 288.7 eV are assigned to C=C and C—N species in N‐carbon. In N Kedge, the resonances of π* at 398.6 and 401.5 eV are assigned to nitrogen species in the form of pyridine (C—N(p)) and graphite (C—N(g)) structures in N‐carbon. The resonances of π* at 399.7 and 402.6 eV are assigned to the aromatic C—N−C coordination of tri‐s‐triazine and the N—3C bridging among three tri‐s‐triazine moieties (C—N(b)) in g‐C_3_N_4_. e) HER polarization curves and f) corresponding Tafel plots of the Ru/C_3_N_4_/C, conventional Ru/C, and commercial Pt/C electrocatalysts recorded in N_2_‐purged 0.1 m KOH solutions. The dashed lines in panels are a guide for the eye to calculate j_0_ by the linear fitting of Tafel plots. In panel (a), the under potential hydrogen adsorption effect in the case of precious metals and the capacitance effect in the case of nanocarbons make that the current start points are not zero. Reproduced with permission.^44^ Copyright 2016, American Chemical Society.

Other noble metals, like Ru,^70^ Au,^20^, ^22^ Ir,^22^ Pd,^20^ Ag,^34^ and their alloys with low‐cost transition metals or doped with nonmetal elements were also studied, leading to significant improvements in HER activities in alkaline medium due to their appropriate atomic and electronic structure and the abundance of exposed active sites.^34^, ^71^, ^72^, ^73^ As a cheaper alternative to Pt, Ru possesses a similar H‐bond energy (≈65 kcal mol^−1^) to Pt, but less studied as a viable HER catalyst.^74^ Recently, an effort has been put forward to improve the activity of Ru in alkaline medium by decorating the Ru NPs on nitrogenated holey 2D carbon structure (Ru@C_2_N).^18^ The Ru@C_2_N electrocatalyst exhibited high TOF at 25 mV (0.75 H_2_ s^−1^ in 1.0 m KOH solution), low overpotentials at 10 mA cm^−2^ (17.0 mV in 1.0 m KOH solution) as well as superior stability. It is worth mentioning that recently reported anomalously structured Ru/C_3_N_4_/C catalyst also showed higher hydrogen generation rate than Pt and have been listed as the best HER electrocatalysts in alkaline medium.^44^ By structural identification, the Ru/C_3_N_4_/C catalyst shows a homogeneous dispersion of Ru NPs with an average size of 2 nm on g‐C_3_N_4_/C achieved by strong interaction between Ru and g‐C_3_N_4_/C phase. The g‐C_3_N_4_ act as a facilitator that promotes the evolution of anomalous fcc crystalline Ru structure while only the sole hcp phase exists when Ru was decorated on carbon. This role of g‐C_3_N_4_ in developing a [110]_f_/[112̅0]_h_ intergrown hcp/fcc phase sharing the (0001)_h_/(111)_f_ interface is also predicted by the theoretical studies and confirmed experimentally as shown in **Figure** **5**a,b. Further near edge X‐ray absorption fine structure (NEXAFS) studies have explored a new nitrogen resonances as a results of Ru interaction with bridging N−3C species of g‐C_3_N_4_ having different coordination number as shown in Figure 5c,d. Thus, nitrogen atoms accept additional charges from Ru atom, consequently a negative shift is observed in the photon energy profile of nitrogen which results in improved activity. The polarization curves of three electrocatalysts recorded in 0.1 m KOH solution show an increase in the HER activity based on overpotential in the following order: Ru/C < Pt/C < Ru/C_3_N_4_/C (Figure 5e,f). Theoretical adhesion energy calculations by DFT agree with the experimental electrocatalytic activity showing that anomalous fcc crystalline Ru structure attributed to the high HER activity, as only a sole hcp phase exist in the Ru/C control sample. Therefore, this study opens a new avenue for the designing a special kind of carbon‐based metallic catalysts to induce an anomalous crystalline structure with high HER activity.

In summary, a great deal of noble metal based efficient and stable nanostructured electrocatalysts have been tailored in attempt to developing ideal HER catalysts in alkaline medium. However, the high cost and scarcity of noble metals are still the major hurdles in commercialization on a mass scale. As an example, Ru is considered low cost but its availability is also 4 times lower than the Pt on earth curst. Thus, keeping in mind the cost issue and scarcity, the development of cost‐effective catalysts based on non‐noble metals or metal‐free catalysts can provide the ideal way to overcome these issues and find a balance between cost and performance.

### 5.2 Non‐Noble Metals and Their Heterogeneous Nanostructures

Very recently, the HER catalysts with earth‐abundant elements, especially cost‐effective transition metals (e.g., Fe, Co, Ni, Mo, W, etc.) and nonmetal elements (e.g., C, N, S, O, P, etc.) have been widely explored in alkaline medium to replace the noble metal catalysts. Noteworthy, alkaline medium also offers an active platform for the materials which are not attractive enough due to their poor stability in acidic mediums, such as pure transition metals,^75^ alloys,^76^, ^77^, ^78^ oxides,^27^, ^79^ and hydroxides^80^ based catalyst materials. Before going to summarize the recent development on non‐noble metals, here first we will list out the strategies adopted for enhancing their HER performances in alkaline mediums.

(i) *Chemical Compositions*: It is considered as a normal but critical strategy for designing high‐performance non‐noble metal catalysts. The chemical compositions, such as the type of elements and the ratios between metal and nonmetal elements, as well as ratios of metallic elements in binary or ternary alloys, influence the electrochemical activity strongly, which has been evidenced by a bunch of articles.^11^, ^81^, ^82^ Taking MoP as an example, MoP owns a better performance compared to Mo_3_P and Mo, due to a higher degree of phosphorization.^18^ Similarly, among the Co_2_P and CoP, the later one perform better as higher P assist in better H‐binding energy.^83^ In fact the P atoms are responsible for creating a high numbers of edges for HER and are verified as the active site for the almost zero Gibbs free energy by DFT calculations. In conclusion, it is the different ratios of P that transform poorly active Mo metal into excellent HER electrocatalysts and results in different HER activities.^84^ Thus, composition is one of the most important factor that can assist in yielding highly efficient catalysts for alkaline HER with high stability.
(ii) *Surface and Geometric Properties*: The geometric properties (size, conformation, crystallinity, etc.) and electronic structure (d‐band center position, work functions, etc.) of heterogeneous electrocatalysts work together to determine adsorption of intermediates, activation energies, and energy barriers.^24^, ^74^ The surface characters such as porosity of composites, the molecular architecture of active metal moieties on surface and macro morphologies of the whole catalysts also play an important role in catalyst design and structure‐activity function.^74^ In the end, the surface chemistry and geometric properties influence the surface reaction rate and the HER performance. For example, controlled amount of CoP nanosheets assembly grown on carbon cloth (CC) with rugae morphology exhibit a Tafel slope of 42.6 mV dec^−1^ in 1 m KOH for HER due to a higher double‐layer capacitance and density of active sites, which is attributed to the rugae‐like morphology.^55^
(iii) *Hybrid with Carbonaceous Materials*: It is obvious that good electrical conductivity and homogeneous dispersion of the active catalysts are significant aspect to enhance HER activities.^85^ The poor electrical conductivity results in a voltage drop across the electrode, producing an extra overpotential and leading to lower apparent catalytic activity and consuming more energy.^86^ Besides, excellent dispersion makes full use of exposed active sites on catalysts to participate in electrode reaction and then improve the electrocatalytic efficiency.^24^ Carbon materials, such as carbon nanotubes (CNTs), graphene, graphene oxide (GO), and porous carbon materials extracted from artificial and natural carbon complexes not only increase the dispersity of the active components but also improve the conductivity of the hybrid catalysts due to their huge surface area, tunable molecular structures and superior electrical conductivity.^51^, ^87^, ^88^, ^89^ Typically, Co‐CNTs was designed as a highly active and stable electrocatalyst which is composed of Co embedded in nitrogen‐rich carbon nanotubes.^90^ The Co‐CNTs showed only marginally lower onset potential by −0.05 to −0.1 V compared to Pt/C and delivers higher current density at slightly higher applied potentials than their onset potential (←0.5 V vs RHE) under the same condition in 1 m KOH, resulting in a better HER activity than the commercial Pt/C. Furthermore, these carbon atoms become active sites by doping higher electronegative element, e.g., N, which render higher positive charge density on their adjacent carbon atoms.^91^ Facile electron transferring from Co NPs inside the CNTs to the surface also contributed to decreased local work function on the carbon surface, thus assist in faster HER kinetics.
(iv) *Heteroatom Doping*: Doping with metal or nonmetal elements is one of the most effective ways to improve electrocatalytic activity. So far, lots of binary or ternary alloys and nonmetal doped electrocatalysts have been tailored with high performances.^5^, ^51^, ^76^, ^92^, ^93^, ^94^, ^95^ Ni‐based binary compounds with different dopants as Ni_3_S_2_, Ni_3_N, or Ni_5_P_4_ show that heteroatoms such as P, S, and N actively take part in the reaction instead as simple spectator.^92^ By correlation of theoretical calculations and electrochemical testing, it was established that the anisotropic nature of the materials result in a general shift in hydrogen adsorption free energies Δ*G*_H_ close to zero for a variety of adsorption sites with highly coverage‐dependent properties.^34^ Thus, the doping of heteroatoms results moderate adsorption energies and introduce bifunctional synergy to assist the faster water dissociation to yield enough amount of protons to accelerate the hydrogen production.^5^ However, the selection of heteroatoms depends on the inherent properties of dopant and host as well their interactions.^96^ Further the contents of heteroatoms in host lattices also largely affect the final properties of hybrid catalysts, so to come up with excellent activity and better stability one should select appropriate dopant and critically analyze required concentration to alter the surface chemistry for better catalysis.
(v) *3D Self‐Supported Nanostructures*: Normally to paste and stabilize the catalyst ink on the commonly used glassy carbon electrode requires electrically insulating polymeric binders, thus forcing to use the inactive carbon to enhance the conductivity by sacrificing the performance.^48^ Further pasting a catalyst on working electrode results in poor connections among the active catalysts and working electrode brings high charge transfer resistance and fall over of sample with passage of time.^33^ In contrast, the 3D self‐supported electrodes can greatly increase the current density by nearly 5–6 times at the same overpotential compared to traditional electrocatalysts coated on 2D substrates by spin‐coating, dip‐coating, or sputtering method.^24^, ^97^ The reasons can be concluded as followings: (1) Commonly 3D current collectors like CC, titanium plate, copper and nickel foam, possess excellent conductivity;^98^ (2) Robust skeleton and large surface increase effectively the contact area of active surfaces to electrolyte.^99^ (3) In situ growth on 3D current collectors and do not require polymer binders (such as nafion, polytetrafluoroethylene) to combine electrocatalysts and substrates.^100^ Polymer binders lead to the higher series resistance, block the active sites and inhibit the diffusion.^101^ Thus, the development of active materials in 3D morphology directly on the current collectors/working electrodes will assist the researchers to improve their HER kinetics.

Herein, we will summarize the recent efforts to develop the non‐noble metal based alkaline medium active HER catalysts by utilizing above mentioned strategies. Among various transition metals, few are more prominent due to their high activity, e.g., Ni‐,^78^, ^95^, ^102^ Co‐,^79^, ^93^, ^103^ Fe‐, Mn‐104, 105 Mo‐, and W‐based catalysts.^57^, ^106^ To make the discussion simpler and for easy comparison, these materials is divided in to two classes, i.e., single‐metal and mixed‐metal catalysts. Further analysis is drawn from the perspective of the above mentioned regulation strategies, their special heterogeneous structures and synthesis methods.

#### 5.2.1 Single‐Metals Based Catalysts

Nickel‐based catalysts are most extensively studied due to their excellent resistance to corrosion in hot concentrated alkaline medium. Ni‐based catalysts are perceived as the most promising electrode materials for HER due to their ability to carry out the hydrogen evolution in alkaline medium because of the special 3d electronic distribution.^35^ As nanostructures can offer significantly higher surface area to bring more exposed active sites and modified the electronic properties, the nanosized Ni‐based catalysts with a uniform size and morphological distributions have been explored to improve the HER activities in alkaline medium.^104^, ^107^ Simply by designing the nanosheets of NiP*_x_*, its activity for HER can be enhanced compared to bulk NiP.^108^ Recently, nickel NPs with different morphologies were synthesized by simple solvothermal reactions using different oleylamine (OAm)/Ni‐source molar ratios in various types of alcoholic solvents. The NiEt‐OAm‐4 with urchin‐like morphology was much more active than others, with an exchange current density of 0.191 mA cm^−2^ as well as long term stability exceeding electrocatalytic performance of a commercial Pt catalysts (40% Pt on Vulcan XC 72).^75^ The higher catalytic performance was attributed to the higher exposure of active sites on catalyst surface based on the special opened morphology and interconnected network for better charge transfer.

Apart from Ni‐based catalysts, Co‐based catalysts (such as metallic Co and its oxides, phosphides, and sulfides), especially cobalt phosphides have attracted extensive attention as novel and efficient HER electrocatalysts for alkaline electrolytes due to their superb electrical conductivity and good durability.^55^, ^103^ As an example, when 3D porous CoP_3_ concave polyhedrons were extracted from metal‐organic frameworks (MOFs), an excellent electrocatalytic activity was observed with better durability in alkaline medium.^103^ Thus, designing open porous structure brings an exciting advancement in developing highly efficient electrocatalysts for alkaline HER. Structural and electronic tuning of Co‐based catalyst is another alternative approach such a Janus like asymmetric NPs to alter the electronic distribution at the interface to boost their catalytic properties. Chen and co‐workers have developed the CoCoP Jnaus like NPs and found that such a structural design can enhance the activity of Co to large extent by attaining the current density of 10 mA cm^−2^ at the overpotential of 253 mV that is ≈2 times lower than pure Co.^23^ Similarly, Zou and co‐workers have introduced a bottom‐up strategy for synthesis of CoP architecture encapsulated into boron and nitrogen co‐doped carbon (BCN) nanotubes (CoP@BCN) through pyrolysis of polymeric subunits and MOF precursors and subsequent phosphidation.^56^ CoP@BCN shows an overpotential of 215 mV at a current density of −10 mA cm^−2^ compared to Co@BCN which requires 260 mV to reach the same current density. The excellent activity of CoP@BCN toward HER is attributed to the following synergistic effects: (i) The metals centers acts as active sites during the hydrogenases, (ii) A charge separation occurs by transferring the electrons from Co to P with support of BCN backbone, and (iii) heterodoped graphitic backbone especially in 1D shape can effectively lower the charge transfer resistance and consequently faster the HER kinetics.^109^ Possibly the P atoms may also act as proton carrier to further accelerate the kinetics of protons (H^+^) adsorption and recombination and can dramatically lower the overpotential for HER.^110^ Further distribution of positively and negatively charge density due to the existence of heteroatoms may make it active toward hydrogen generation.^91^, ^111^ Taking advantage of heteroatom doping on conductivity and enhancing the intrinsic activity of CoP, Zou and co‐workers have developed N–P co‐doped carbon matrix for CoP to eliminate the inactive carbon addition to catalyst ink and improve the activity.^112^ They have beautifully pinned the CoP particles to carbon matrix through P and N atoms, which create strong attraction and inhibit their aggregation during electrochemical testing. Further these covalent bonding also alter the electronic structure of CoP, results in enhanced current density (10 mA cm^−2^) at lower over potential of 120 mV.

**Figure 6:**
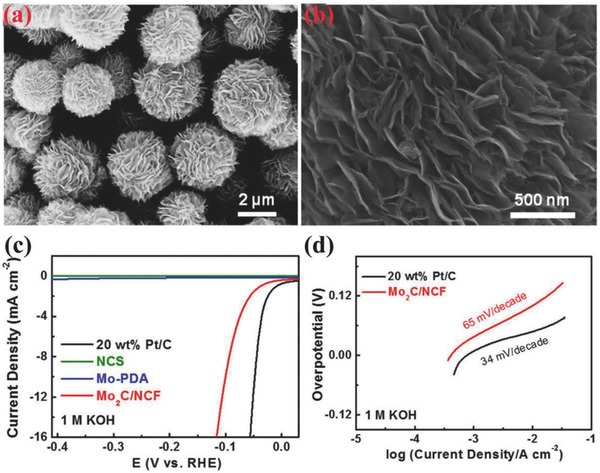
a,b) SEM images at different magnifications; c) polarization curves, and d) corresponding Tafel plots of Mo_2_C/NCF in comparison with 20 wt% Pt/C benchmark, Mo‐free NCS, and unpyrolyzed Mo‐PDA in 1M KOH. Reproduced with permission.^99^ Copyright 2016, American Chemical Society.

In the past decades, a great interest has been shown to investigate about Mo and W based catalysts, although these metals have been extensively studied for acidic HER catalysis but have very limited activity in the alkaline electrolytes.^113^, ^114^, ^115^ However, their carbides, sulfides and phosphides have been explored recently with good activity toward HER in alkaline electrolytes.^57^, ^106^, ^116^, ^117^, ^118^, ^119^, ^120^ As molybdenum carbides own the electronic structure similar to that of Pt and possess high resistance to corrosion in alkaline medium, it has attracted extensive research attention as efficient HER electrocatalysts,^57^ even though these catalysts need relatively large overpotential of 190–230 mV to achieve the appropriate current densities.^99^ In order to enhance the activity of Mo_2_C, its NPs were dispersed on hierarchical carbon microflowers (Mo_2_C/NCF) via a facile two‐step preparation method through the self‐polymerization of dopamine (**Figure** **6**a,b).^99^ In 1 m KOH solution, the HER activity of Mo_2_C/NCF is enhanced and its overpotentials to achieve 1 and 10 mA cm^−2^ is reduced to 38 and 100 mV, respectively (Figure 6c,d).^99^ Furthermore, the most important advantage of Mo in alloy or composite structures is to bring high stability and low overpotential.^87^ The new class of MoS_2_@Ni core/shell nanosheets array vertically aligned on carbon cloth (MoS_2_@Ni/CC) showed excellent HER activity in alkaline medium, with overpotentials of 91, 118, and 196 mV to approach current densities of 10, 20, and 100 mA cm^−2^, respectively.^106^ The enhanced activities are presumably contributed to the synergistic effects between MoS_2_ and Ni.^106^ Further, by expanding the interlayer distance and edge alignment of MoS_2_ nanosheets can also boost up its activity toward HER in various electrolytes.

**Figure 7:**
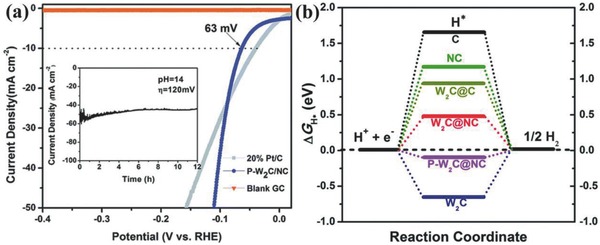
a) Polarization curves of P‐W_2_C@NC after *iR* correction in 1 m KOH (inset: time dependence of the HER current density of P‐W_2_C@NC at a static overpotential of 120 mV for 12 h). b) The calculated free‐energy diagram of the HER on various catalysts; the graphene shows a large Δ*G*(H*) value of 1.832 eV, indicating a negligible adsorption ability of H*. NC shows a positive Δ*G*(H*) value (1.181 eV), representing a low HER activity. P‐W_2_C@NC gave a much smaller Δ*G*(H*) value (−0.112 eV) than its constituents (i.e., W_2_C, W_2_C@NC, NC, and C), indicating that P and N dopants in P‐W_2_C@NC can reduce the value of Δ*G*(H*) and enhance the initial H* adsorption. Reproduced with permission.^117^ Copyright 2017, Royal Society of Chemistry.

Similarly, Chen and co‐workers prepared the polymorphic tungsten diphosphide (p‐WP_2_) NPs having mixed monoclinic (α‐) and orthorhombic (β‐) phases through a phase‐controlled phosphidation method.^31^ The p‐WP_2_ catalyst delivered excellent HER activity in 1 m KOH by achieving the current density of 10 mA cm^−2^ at an overpotential of 175 mV, far better than α‐WP_2_ (required 259 mV) and β‐WP_2_ (required 277 mV).^31^ Further, p‐WP_2_ catalyst also showed high stability by delivering almost same performance after 1000 cyclic voltammetry sweeps. Thus, the superior performance of p‐WP_2_ proved that by controlling the extent of different phases of tungsten phosphides in single particle can open the possibilities to tune its activity toward HER catalysis. Although the reason behind such enhancement is unclear yet, the distortion in lattice structure at interface may play an effective role in tuning its electronic properties that strongly affects the activity of any catalyst. Therefore, the structural analysis of p‐WP_2_ confirms the lattice distortion as mismatching of the lattice constant of α‐WP_2_ and β‐WP_2_ phases is observed, which significantly reduces the Gibbs free energy for hydrogen evolution. Besides the defective approach, doping of heteroatom like P can also be very effective way to tune the electronic structure and surface chemistry of W‐based materials such as tungsten carbide (WC) to enhance the alkaline HER.^117^ Although WC carbide has been explored a lot as catalyst for various important reaction even for HER due to its Pt‐like d‐band structure, however, its traditional synthesis approaches does not activate its inherent catalytic properties toward HER, especially in alkaline electrolytes where it is highly unstable.^7^, ^120^ Polyoxometalates (POMs) having a TM center with uniform size and well‐defined structures, provides an opportunity to extract highly fascinating materials for various application with protected shells extracted from functional carbon frame work of POMs.^121^ Taking advantage of special nanoscale features and better dispersion of POMs, small size WC*_x_* can be prepared to enhance its activity in KOH solution. However, to realize the dream of nanomaterials extraction from POMs depends on their dispersion that is one of the critical step. Recently, Li and co‐workers have put their efforts to extract p‐doped WC (P‐WC*_x_*) from W‐based POM clusters.^117^ For this purpose, the mixture of H_3_PW_12_O_40_ (PW_12_) and dicyandiamide (DCA) was heated up to 400 °C in N_2_ atmosphere. The CN*_x_* fragments were employed to separate the PW_12_ clusters by preventing their agglomeration during carbonization process to prepare WC*_x_* NPs. Additionally, these CN*_x_* fragments also provides the opportunity to construct a thin protective overcoat on the surface of WC*_x_* to enhance its stability in alkaline medium. Also the carbon contents can be adjusted by the initial POM and DCA amounts which also affect the overall surface area of hybrid. In addition the P and N atoms in POMs and CN*_x_* fragments helps to modify the electronic structure of WC*_x_* and surface chemistry of hybrid, respectively, which can promote its HER catalytic activity. Taking these structural and compositional advantages, the P‐W_2_C@NC hybrid perform excellently in 1 m KOH as delineated from its polarization curve, a very small overpotential of 63 mV is required to reach the current density of 10 mA cm^−2^, only 25 mV larger than commercial 20% Pt/C (**Figure** **7**a). Although the P‐W_2_C@NC show very good activity compared to the many other reported materials due to its unique structure and composition, however, it still suffered from poor stability as shown by the *i*–*t* test, a 13% decrease in the current density over 12 h of test (the inset of Figure 7a). The better activity of P‐W_2_C@NC goes to its surface protection which prevents its surface oxidation and doping of heteroatoms that lowers its H adsorption energy as confirmed by the DFT calculation presented in Figure 7b.

#### 5.2.2 Mixed‐Metal Based Catalysts

So far, high‐surface‐area Raney‐type designing of Ni‐based alloys as well as synthesis of heterostructured Ni‐based compounds with unique composition to modify the surface chemistry have attracted more attentions.^88^, ^122^, ^123^, ^124^ The properties of Ni surface can be modified by additional elements to improve its activity. A nearby heteroatom may alter surface adsorption/desorption energy on adjacent Ni atoms and serve as adsorption/desorption sites for certain catalytic intermediate species.^100^ From this aspect, Ni‐based compounds with nonmetal elements were investigated to improve the activity of Ni for HER in alkaline medium.^78^, ^92^, ^102^ Among abundant interfaces, multicomponent heterostructures can maximize the direct interfacial contact between metal atoms which significantly enhances the electron transfer and shorten the diffusion distances, while the synergy among these components can result in enhanced activities.^11^, ^95^, ^125^ To explore the activity of Ni‐based compounds, Hu and co‐workers prepared self‐supported NiP*_x_* and Co‐doped NiCo_2_P*_x_* nanostructures on carbon felt (CF) using wet chemical–hydrothermal methods coupled with in situ thermal phosphorization process.^45^ It was observed that NiP*_x_* delivered an onset potential of 89 mV (normally consider as the potential at current density of 1 mA cm^−2^), but interestingly the incorporation of Co leads to very low onset potential of 11 mV (NiCo_2_P*_x_*).^45^ Further, to achieve the current density of 10 mA cm^−2^, the NiP*_x_* required an overpotential of 180 mV that is also reduced to only 58 mV in case of NiCo_2_P*_x_* which once again shows superior performance of NiCo_2_P*_x_* even better than the Pt catalyst (70 mV).^45^ It is also worth mentioning that such a heterostructured designing of NiCo_2_P*_x_* is highly fascinating in driving the high current density values up to 100 mA cm^−2^ at very low overpotential of 127 mV, making it highly suitable for industrial applications.

**Figure 8:**
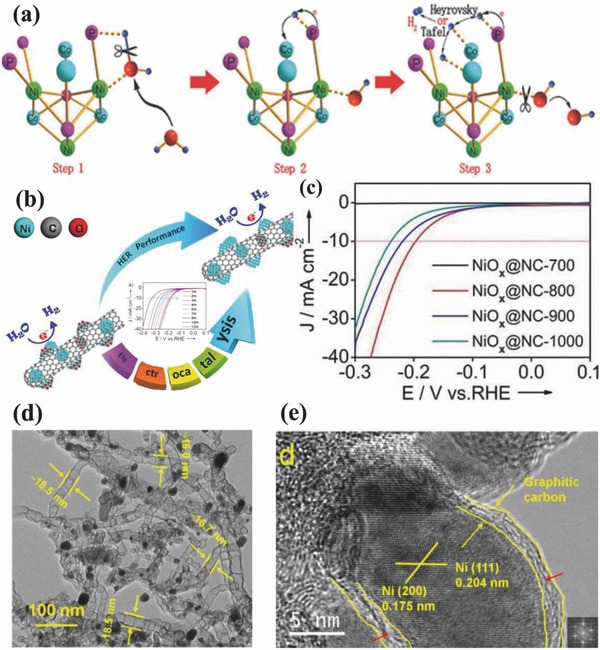
a) Schematic illustration of water dissociation process in alkaline solutions on NiCo_2_P*_x_* surface. Reproduced with permission.^45^ b) Schematic illustration of bamboo‐like NiO*_x_*@BCNTs. c) Polarization curves for samples calcined at different temperature in 0.1 m KOH solutions. d) TEM image of NiO*_x_*@BCNTs treated with acid; e) HRTEM images of NiO*_x_*@BCNTs. Inset in part (e) is the fast Fourier transform (FFT) images of Ni^o^. Reproduced with permission.^126^ Copyright 2016, American Chemical Society.

It is well‐known that HER process in alkaline medium is governed by the dissociation of water molecules, where two intermediate species forms, i.e., OH^−^ and H^+^ which needs to adsorb and desorb on catalyst surface with variable deriving energies to complete the reaction. The literature has demonstrated that Ni is preferable site for desorption of OH^−^ compared to Co in alkaline solutions, on the other hand Co performs better in the Heyrovsky and Tafel process. Therefore in case of NiCo_2_P*_x_*, Ni active sites at surface are involved in the water dissociation with high turnover frequencies while the Co sites will actively involve in the generation and release of hydrogen. Such a working behavior of Ni and Co in NiCo_2_P*_x_* is similar to the phenomenon observed in case of Ni(OH)_2_/Pt(111) catalyst explained above. Based on above discussion, the mechanism of NiCo‐based compounds and their synergistic effect on HER in alkaline electrolytes can be explained in three steps as shown in **Figure** **8**a. In 1st step: an electrical double layer (EDL) formed on the surface of NiCo_2_P*_x_* by water molecules, where electronic effects will weaken the H—OH bond. The H—OH bonds is weekend by the interactions among the under coordinated metals sites (M*^δ^* ^+^, where M is Ni, Co) and oxygen atoms while dangling P atoms (P*^δ^* ^−^) contribute by attracting the hydrogen atoms. In 2nd step: the dissociation of water molecules occurs with the support of free electrons in to H^+^ and OH^−^. In the final 3rd step: H atoms adsorbed on the vacant metal sites next to the one which is covered by the OH^−^ and denoted as adsorbed H. Therefore, the entire alkaline HER process depends on the kinetics of recombination of adsorbed H atoms and desorption of OH^−^.

Carbon can also act as very good support and dispersant for active NPs to prevent their aggregation and provide more exposed surface area. Yao and co‐workers prepared 2D heterostructure like Ni–Fe‐layered double hydroxide (LDH)‐NS@DG hybrid catalysts by homogeneously stacking of the exfoliated Fe–Ni‐LDH cation sheets onto the negatively charged defective graphene (DG).^80^ The as‐prepared hybrid nanostructures exhibited good HER performance with a overpotential of 300 mV at 10 mA cm^−2^ and a small Tafel slope (110 mV dec^−1^), which is ascribed to the enhanced density of electron transfer at specific domains caused by the defective sites on DG. Currently, heteroatom‐doped graphitic carbon materials have been applied in HER as well because of their large specific surface area and unique electronic properties.^126^ Bamboo‐like nitrogen‐doped carbon nanotube based nickel oxide (NiO*_x_*@BCNT) hybrid was designed through calcination of melamine/Ni(NO_3_)_2_·6H_2_O (Figure 8b,c). Excellent catalytic activity is shown with an overpotential of 79 mV at a current density of 10 mA cm^−2^ and considerable durability is obtained by adjusting the mass ratio of the precursors and the pyrolytic program (Figure 8d). The experimental results and DFT calculations showed that both bulk Ni and Ni on the interface of Ni/NiO contributed to the outstanding catalytic performance. Besides, the in situ generated Ni from NiO during the HER process also accelerated the hydrogen generation kinetics.

**Figure 9:**
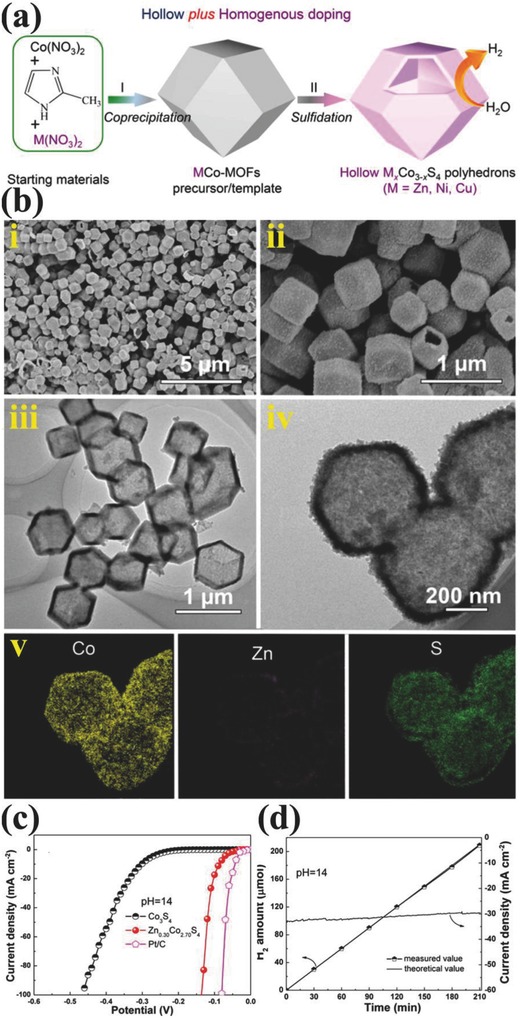
a) Schematic illustration of fabrication of hollow Co‐based bimetallic sulfide. b) SEM (i and ii), TEM (iii and iv) images, and (v) elemental maps of Zn_0.30_Co_2.70_S_4_. c) Polarization data of Zn_0.30_Co_2.70_S_4,_ Co_3_S_4_ and Pt/C electrodes at pH = 14. d) Electrocatalytic hydrogen production over Zn_0.30_Co_2.70_S_4_ at pH = 14. Reproduced with permission.^127^ Copyright 2016, American Chemical Society.

The lower energy barrier for H adsorption of cobalt‐based materials make them promising catalysts for HER, which urges many researchers to develop various cobalt based nanomaterials.^16^ Taking the advantage of H adsorption energy of Co, Zou and co‐workers reported the hollow Co‐based bimetallic sulfide (M*_x_*Co_3−_ *_x_*S_4,_ M = Zn, Ni, and Cu) polyhedra by a simple self‐template strategy.^127^ Simply homogenous bimetallic MOFs were transformed to hollow bimetallic sulfides by solvothermal sulfidation and thermal annealing. Among various bimetallic catalysts, the Zn_0.30_Co_2.70_S_4_ exhibited superior HER activity (overpotential of 85 mV at 10 mA cm^−2^ in 1 m KOH solution) and stability because of the optimized Gibbs free energy for H* adsorption and improved electrical conductivity due to the homogeneous distribution of Zn in Co_3_S_4_ lattice as shown in **Figure** **9**.^127^ This study also showed that composition plays effective role in optimizing the Gibbs free energy for H* adsorption, while the morphology tunes the exposer of active sites. It is also found that hollow structure can minimize the mass transfer resistance to enhance the reaction kinetics. Composite formation of two different materials to overcome their limitations is another fascinating technique, where Chen and co‐workers designed RuO_2_/Co_3_O_4_ heterojunction by directly annealing a MOF derived Co–Ru complex under atmospheric conditions.^79^ The catalyst showed a lower HER overpotential (89 mV at 10 mA cm^−2^ in 1 m KOH solution) and outstanding long‐time durability, which indicated that a heterojunction created by transition metal oxides with small amounts of noble metal oxides can provide more active sites for high electrocatalytic performance, with assistance of efficient water dissociation by Ir.^79^

Recently, coupling of Co‐based active materials with carbon nanostructures and 3D self‐supported electrodes are of immense importance and widely explored. Such as Feng et al. reported Co nanotubes decorated with TiO_2_ nanodots (NDS;NSNTs) supported on carbon fibers (TiO_2_‐NDs@Co‐NSNTs/CFs) as low cost but high‐performance electrocatalysts for HER in alkaline medium.^128^ The special electronic interactions between TiO_2_ and Co can change the surface electronic state of electrocatalysts and accordingly can activate the absorbed water molecules as well as optimize the free energy of hydrogen adsorption, which obviously promote HER kinetics.^128^ It has been proved that the heteroatoms doping having variable electronegativity or/and size is effective way to modify the electronic structure and surface chemistry of carbon.^9^, ^10^, ^129^ Therefore, Wu and co‐workers developed a simple pyrolysis approach to prepare nitrogen, oxygen, and sulfur tridoped carbon encapsulated Co_9_S_8_ (Co_9_S_8_@NOSC) nanomaterials using S‐ and Co(II)‐containing polypyrrole solid precursors, which has very high affinity for water oxidation.^130^ In contrast to bimetallic oxides, phosphides, and sulfides of Co, its selenides are also very active toward alkaline‐HER. As an example, Zhang and co‐workers prepared an integrated electrocatalyst composed of 3D Ni_1−_ *_x_*Co*_x_*Se_2_ mesoporous nanosheets network on nickel foam (NF) as substrate for direct use with tunable stoichiometry by the simple selenization method of NiCo‐LDH nanosheets arrays.^48^ An acid treatment was employed after selenization to create the mesoporosity and tune the surface chemistry to deliver low overpotential of 85 mV to achieve the current density of −10 mA cm^−2^ in 1 m KOH. The DFT studies have verified that Ni_0.89_Co_0.11_Se_2_ possess the metallic states which have a high electrical conductivity, while existence of Co in the lattices of Ni_0.89_Co_0.11_Se_2_ in an optimal amount modified the adsorption of both H^+^ and H_2_O on Ni_0.89_Co_0.11_Se_2_ surfaces with appropriate free energies. Thus, Co is responsible for enhancing the HER catalytic activity along with excellent stability.

Compared to Co and Ni elements, Fe takes the advantage of its low price and high abundance in the earth crust but there are very limited reports on Fe‐based HER electrocatalysts compared with Co and Ni‐based materials. Recently, Fe based catalysts have been tailored to modify the electronic structure which has opened up the ways for Fe as an effective catalyst for alkaline HER. For example, Chen and co‐workers prepared the NiFeP by simple phosphorization of NiFeO*_x_* and achieve the current density of 200 mA cm^−2^ at overpotential of 220 mV with low values of Tafel slop (78 mV dec^−1^).^131^ An iron foam was applied as a 3D current collector substrate which provided an intimate contact and high wettability to deliver excellent activity. In another report, microwave‐electrochemical deposition of a Fe–Co alloy showed low overpotential of (145 mV), high exchange current density (20 mA cm^−2^) and Tafel parameters close to those for Pt on stainless steel.^104^ Use of stainless steel as support and current collector suggested that in the future microwave‐electrochemical deposition method can help in providing novel sustainable alloy catalyst materials for a wider range of applications.^104^ To take the advantage from the performance enhancing activities of carbon nanosheets, Guo and co‐workers prepared the FeCo alloy on the carbon nanosheets in 3D morphology to expose more active sites and lower the onset potential of FeCo alloy to only −63 mV.^33^ It was also explored that the amounts of Fe and Co in resulting alloy have a strong impact on the activity of final alloy, thus an appropriate control over composition is necessary. Sun and co‐workers reported Fe‐doped CoP nanoarray on Ti foil (Fe‐CoP/Ti) with high HER activity superior to CoP nanoarray, outperforming most of reported non noble‐metal catalysts, with an extremely low overpotential of 78 mV for 10 mA cm^−2^ in 1 m KOH.^105^ Therefore, these studies open new opportunities in designing and utilizing monolithically integrated ternary or doped transition metal phosphides as well as their other compounds in various structure, e.g., nanoarrays as cost‐effective and robust multifunctional materials for practical applications.^105^

Although the 3d TM metals show good activity toward water oxidation, however Mn is not so good for hydrogen catalysis directly. In contrast to use solely as HER catalyst, Mn exhibits very high activity toward water electrolysis that is why Mn is extensively studied for oxygen evolution and reduction reactions.^8^, ^37^ Considering its high affinity for water dissociation and requirement for alkaline HER catalysis, Yang and co‐workers developed bifunctional catalyst in alkaline electrolytes by introducing Mn_3_O_4_ in metallic Ni through hydrothermal reaction on the NF.^37^ Interestingly, the resulting Ni‐Mn_3_O_4_/NF hybrid catalyst delivered excellent activity and stability toward HER by reaching the current density of 10 mA cm^−2^ only at very low overpotential of 10 mV in 1 m KOH. The activity and good stability of Ni‐Mn_3_O_4_/NF hybrid is based on the bifunctional effect of Mn_3_O_4_ that accelerate the water dissociation and produce hydrogen intermediates while Ni adsorb the hydrogen intermediates and recombine them to molecular hydrogen. Furthermore, the Mn_3_O_4_ increases the metallic characteristics of Ni which greatly influence the charge transport among the electrode and electrolyte. The introduction of bifunctionality through hybridization of two materials opens a new way in designing efficient catalyst materials for alkaline HER especially by overcoming the additional barrier of water dissociation.^34^, ^71^ Recently, heterogeneous nanostructures of CoS_2_@WS_2_/CC have been developed to create unique nanointerfaces through the intermediator sulfur atom (Co–S–W), resulting in high activity as required only 97 mV to achieve current density of 10 mA cm^−2^.^119^ This strategy presents a unique method to tune the interface to activate the active sites and enhance the activities of catalysts.^132^

### 5.3 Nonmetal Electrocatalysts

Efficient and low‐cost nonmetal electrocatalysts mostly known as carbon based materials for the HER are highly desired for future renewable energy systems to get rid of high cost and expensive metallic counter parts.^97^ Further, carbon based materials provide great opportunities to play with morphology and surface chemistry, while both of these factors play key role in exposing and stabilizing the active sites for efficient catalysis.^91^, ^133^, ^134^, ^135^ Carbon based materials also provides additional stability in alkaline electrolyte due to their oxidation resistive nature compared to metal based catalysts.^30^ Although, carbon based materials have several advantages over metallic catalysts but there is a very limited work on carbon based catalysts especially for alkaline HER. The reason behind this less exploration of carbon based materials as an alternative catalysts is their poor capability of water oxidation and simultaneous adsorption and recombination of proton to evolve hydrogen. However, by controlling the sp^2^ and sp^3^ hybridization degree the catalytic properties of the carbon based materials can be tuned. Further the incorporation of heteroatoms (metallic or nonmetallic elements) in the lattice planes of carbon nanostructure is also widely used to mobilize the electronic movements and break the surface neutrality, especially in case of graphitic carbon where heteroatoms can alter the density of state (DOS) of carbon and alter its electronic atmosphere.^90^, ^136^, ^137^ This kind of modification can enhance their abilities for high electrical conductions, surface reactions and active sites for chemical conversions and chemo/physio adsorptions of molecular components. The alloy formation of two nonmetallic atoms likewise carbon with nitrogen in graphitic structure is another opportunity to tune the catalytic activities of nonmetallic elements.^91^ Recently researchers have demonstrated the ability to tailor metal‐free C_3_N_4_ electrocatalyst to reduce water in to hydrogen both in alkaline and neutral environments.^138^ However, the poor conductivity of C_3_N_4_ is still limiting their application in catalysis, thus C_3_N_4_ needs to build a direct connection with conductive materials to improve their efficiencies for electrocatalysis. For example, Antonietti and co‐workers presented a generic, facile, and easy water based method coupled with thermal annealing to grow highly ordered C_3_N_4_ on different substrates from surface‐bound cyanuric acid and melamine supramolecular complexes and demonstrate the high suitability of this material to catalyze the HER.^139^ The metal‐free catalyst generally delivers low overpotential values, which are comparable to those of non‐noble metals while having reasonable current densities. The C_3_N_4_/FTO (fluorine‐doped tin oxide) electrode demonstrated low overpotential ≈0.1 V versus RHE and with current densities of 0.8 mA cm^−2^ at the overpotential of 0.3 V in 0.1 m KOH. The current densities can be increased further by using different types of supportive materials like TiO_2_, ZnO, etc. For example, the C_3_N_4_/TiO_2_ electrocatalyst shows better current density (1.3 mA cm^−2^ at an overpotential of 0.3 V) at same overpotential than C_3_N_4_/FTO. It is concluded that substrate surface chemistry also strongly effect the physical and chemical properties of the catalysts. Such as deposition of C_3_N_4_ on different substrates (as above TiO_2_ and FTO) strongly affects their morphologies, new interfaces, formation of surface defects or new active states as well as alter their electronic and optical properties, resulting in variable catalytic activities. Thus, strategies to tune the catalyst functions based on substrate and changing the composition of starting complex opens the opportunity for a new family of materials for HER.

Although these strategies have proved the active nature of nonmetallic materials for HER, but their current densities are far poor than their metallic counterparts that needs to be improved for their practical applications in real devices. In this regards Xu and co‐workers design a 3D O, N, and P tridoped porous graphitic nanocarbons (ONPPGC) directly grown on oxidized CC (OCC) as a binder free electrocatalyst for alkaline hydrogen catalysis.^140^ The ONPPGC/OCC represents the first carbon nanomaterial based nonmetal class of catalyst for full water splitting, which was prepared by using aniline and phytic acid as precursors and OCC as supportive substrate. In 1 m KOH solution, ONPPGC/OCC exhibited improved activity with an overpotential of 446 mV to achieve the current density of 10 mA cm^−2^. A better value of Tafel slope (154 mV dec^−1^) for ONPPGC/OCC than bare CC (193 mV dec^−1^) proves that designing of heteroatom doped carbon can alter its nature through covalent interaction. Further it is also found that the strong coupling in ONPPGC/OCC also results good electrochemical stability as catalyst only require a potential of ≈0.4 V to deliver 7.6 mA cm^−2^ and current density then stabilized around this value for the 5 h reaction time, unprecedented stability. The activity of ONPPGC/OCC shows that 3D porous electrodes can possess excellent activity and durability for all pH values to achieve water splitting in electrocatalytic system. When using ONPPGC/OCC as both anode and cathode, an efficient basic water electrolyzer demonstrates 10 mA cm^−2^ at a cell voltage of 1.66 V with superior stability, which inspires exploring 3D porous metal‐free electrode for full water splitting.

**Figure 10:**
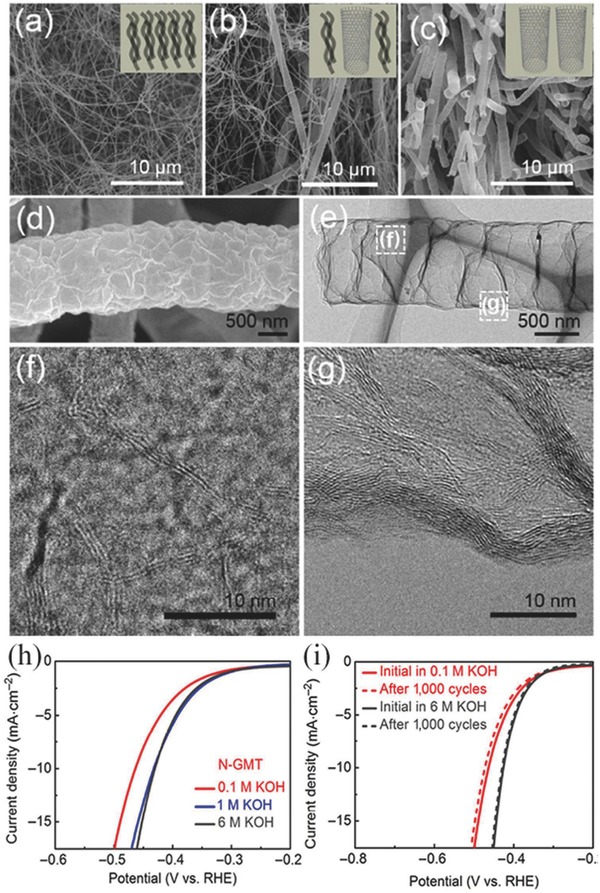
Large‐area SEM images of a) N‐CNT, b) N‐CNT+NGMT, and c) N‐GMT, which are obtained from the same precursor (a 1:40 mixture of glycin and DCDA) at 900, 1,000, and 1100 °C, respectively. Detailed d) SEM, e) TEM, and f–g) HRTEM images of N‐GMT, h) IR‐corrected LSV curves of N‐GMT in basic solution with various KOH concentrations, i) LSV curves of N‐GMT before and after 1000 cycles of cyclic voltammetry in 0.1 and 6 m KOH solution. Scan rate is 10 mV s^−1^. Reproduced with permission.^142^ Copyright 2016, Springer.

Zheng and co‐workers developed a homologous, metal‐free electrocatalyst for HER in alkaline media consisting of self‐supported sulfur‐doped carbon nitride/carbon nanotube/carbon fiber (S‐C_3_N_4_‐CNT‐CF).^141^ The CNTs are deposited in 3D fashion on the interconnected CF networks and thermally treated which make the overall structure highly, enhance the electrical conductivity and result robust mechanical stability. A π–π stacking is utilized for in situ growth of C_3_N_4_ on the surface of CNTs and CFs to form C_3_N_4_‐CNT‐CF hybrid structure which is further sulfurized for sulfur doping to form S‐C_3_N_4_‐CNT‐CF. In 1 m KOH, S‐C_3_N_4_‐CNT‐CF electrode showed an onset potential of 50 mV and an overpotential of 131 mV versus RHE to achieve a current densities of 1 and 10 mA cm^−2^, respectively, with a Tafel slope of 79 mV dec^−1^. Moreover, it exhibited excellent stability for 30 h test duration in 1 m KOH solution, thus suggesting the subsequent sulfur doping along with unique porous structure can improve the HER performance of the C_3_N_4_‐based electrodes. Substitutional doping and the introduction of nitrogen atoms are often used to modify the hexagonal lattice of carbon nanomaterials because pristine sp^2^ carbon is inactive for most electrochemical reactions. However, to expose more easily accessible active centers to the electrolyte for catalysis, more molecules and ions in the electrolyte need to pass through the facial plane of sp^2^ carbons. Therefore, to improve mass transfer efficiency for HER, Zhang and co‐workers prepared nitrogen‐doped carbon microtubes or nitrogen‐doped graphene microtubes with ultrathin walls of 1–4 nm and large inner voids of 1–2 µm to combine the advantages of NG and N‐CNT.^142^ The N‐GMT were prepared through a simple carbonization of a mixture of glycin and dicyandiamide (DCDA) (**Figure** **10**a–g). Here, nitrogen doping in GMT provided more active sites, resulting in significantly enhanced HER activity with a current density of 10 mA cm^−2^ at overpotentials of 0.464 and 0.426 V in 0.1 and 6 m KOH solution, respectively (Figure 10h,i). To the best of our knowledge, this HER performance is the best in basic solution among the metal‐free catalyst reported till now. The N‐GMT promises to be an efficient HER catalyst for real applications in water splitting and chlor‐alkali processes. These findings inspire exploiting structure dependent functions to design efficient metal‐free graphene based nanocatalysts for electrochemical applications as well as other liquid phase catalytic reactions. Taking advantage of similar dual heteroatom doping, Qiao and co‐workers developed “graft‐and‐pyrolyze” technique to develop N,S‐codoped carbon materials, a new methodology fully different from older post treatment strategies.^30^ Initially, PDA was deposited on the CNTs surface and then chemical grafting was done by thiol groups as S‐precursors at room temperature. After pyrolyzing at high temperature, the N,S‐CNTs hybrid delivered the current density of 5 mA cm^−2^ at very low over potential of 40 mV in 1 m KOH, outperformed many other carbon based HER catalysts. Hence, proving that surface chemistry of carbon based materials is a key factor in tuning their catalytic abilities.

## 6 Conclusion and Future Perspectives

As one of the most important reactions of water catalysis, HER has gained extensive attention of researchers over the last decade and large number of catalysts have been developed to enhance the HER kinetics. Unfortunately, the HER kinetics and mechanism vary with nature of used electrolytes and becomes more tough as moving from acidic to alkaline one, more sluggish kinetics involve due to additional water dissociation step. This is the one reason why the best performing acidic medium catalysts have very poor activity in alkaline electrolytes, even the noble metals are suffering from poor stability and large overpotentials to achieve reasonable current densities. The mechanism is not as simple as in case of acidic media where only hydrogen binding energies decide the reaction rate, while in alkaline electrolytes water adsorption and its dissociation, hydrogen adsorption/desorption energies and hydroxyl ions affinity to catalyst surface (poisoned the surface by occupying active site) decide the reaction pathway and rate. Thus, sluggish kinetics and poor activities of most of the catalysts limit the development in the alkaline HER catalysis, one of the most industrial and economically demanding hydrogen production systems. To overcome the challenges associated with unclear sluggish reaction mechanism of alkaline HER, recently several efforts have been put forward by combining the two different catalysts. Likewise the decoration of Pt surfaces with Ni(OH)_2_ or introducing Mn_3_O_4_ into Ni metal based catalysts which can first dissociate the water to generate hydrogen intermediates that can be adsorb with nearby active sites for recombination to produce hydrogen. Besides generating simple composite structures that increase the cost, complicated fabrication methods and utilize high price rare metals, designing of low cost TM or nonmetal based catalysts by tuning their compositions to alter the electronic and surface chemistries is a considerably better choice. Such combinations of various metals in their compounds and/or heterostructures with nonmetals provide an excellent way to generate bifunctionality (water dissociation and simultaneous hydrogen recombination) within a single catalyst to boost the hydrogen production in alkaline solutions. In this regard, 3d metals‐based catalysts have shown a promising future by delivering high current densities at low overpotentials, while bears good stability. To take these bifunctional catalysts to next step to improve their efficiencies further, their in situ designing on the highly conductive 3D substrates were carried out. Direct growth on conductive supports can enhance their conductivity and active surface area as well as generate intimate contact with current collector to lower the charge transfer resistance and 3D morphologies expose more active sites and increase the mas transfer, ultimately improve the HER activity. Although, newly developed strategies have shown high potential and make the HER possible in alkaline electrolytes with low overpotential and high stability but still several things need to be considered for stable and economical operations. In fact, the heterostructures take the advantages of unique electronic structure which results in exceptional properties like faster recombination rate, higher electronic mobility, better mass transport pathways, and finally long term stability. Further, heterostructures also provide easy control over their surface tuning to expose the active sites, hence providing the opportunity to utilize maximum material in catalysis that ultimately helps in cost reduction.

First of all the HER mechanism in alkaline electrolyte needs to be clarified using both theoretical studies and experimental results. The DFT calculations can be employed to see how the different surfaces affect the HER catalysis as well as what kind of relation exists among the adsorption/desorption energies of water, proton, and hydroxyl ions and how these affect the final reaction rate. Then to verify these theoretical studies, in situ microscopic, structural, and compositional characterization should be employed, to see how the various surfaces are behaving during the HER catalysis under practical conditions and what factors are controlling the releasing of hydrogen from catalyst surface. It can also reveal that how the catalyst surface is changing during the HER reaction and to what extent it recover. Thus, taking in account all these theoretical and experimental findings, the tuning of advanced catalyst can be done to overcome the challenges associated with the alkaline electrolytes and limit their applications. On the other hand, these findings will also lead the researchers toward developing integrated catalyst with perfectly required surface chemistry, composition, structure, and morphologies that can have the ability to lower the free energies and overpotential with long term stabilities. Besides developing new catalysts or/and modifications of existing catalyst, the cost should be kept in mind as higher cost of catalyst will increase the overall price of resulted hydrogen. The catalysts should be comprised of low‐price metals/nonmetals and their synthesis method should be very facile that can be extended to industrial scale without any modification and can produce large amounts of hydrogen. Apart from the synthesis of catalyst, the hydrogen production systems should be integrated in such a way that they can easily be coupled with the other energy systems for its continuous production, storage, and easy transport to the end users.

## Conflict of Interest

The authors declare no conflict of interest.
